# A genomic perspective on HLA evolution

**DOI:** 10.1007/s00251-017-1017-3

**Published:** 2017-07-07

**Authors:** Diogo Meyer, Vitor R. C. Aguiar, Bárbara D. Bitarello, Débora Y. C. Brandt, Kelly Nunes

**Affiliations:** 1 Department of Genetics and Evolutionary Biology, University of São Paulo, 05508-090 São Paulo, SP Brazil; 2 Present Address: Department of Integrative Biology, University of California, Berkeley, CA USA; 3 Present Address: Department of Evolutionary Genetics, Max Planck Institute for Evolutionary Anthropology, Leipzig, Germany

**Keywords:** HLA (human leukocyte antigen), MHC (major histocompatibility complex), Evolution, Genomics, Balancing selection

## Abstract

Several decades of research have convincingly shown that classical human leukocyte antigen (HLA) loci bear signatures of natural selection. Despite this conclusion, many questions remain regarding the type of selective regime acting on these loci, the time frame at which selection acts, and the functional connections between genetic variability and natural selection. In this review, we argue that genomic datasets, in particular those generated by next-generation sequencing (NGS) at the population scale, are transforming our understanding of HLA evolution. We show that genomewide data can be used to perform robust and powerful tests for selection, capable of identifying both positive and balancing selection at HLA genes. Importantly, these tests have shown that natural selection can be identified at both recent and ancient timescales. We discuss how findings from genomewide association studies impact the evolutionary study of HLA genes, and how genomic data can be used to survey adaptive change involving interaction at multiple loci. We discuss the methodological developments which are necessary to correctly interpret genomic analyses involving the HLA region. These developments include adapting the NGS analysis framework so as to deal with the highly polymorphic HLA data, as well as developing tools and theory to search for signatures of selection, quantify differentiation, and measure admixture within the HLA region. Finally, we show that high throughput analysis of molecular phenotypes for HLA genes—namely transcription levels—is now a feasible approach and can add another dimension to the study of genetic variation.

## Introduction

The availability of genomic data at the scale of populations is transforming our understanding of the processes shaping human genetic variation. We are now able to answer questions which, little more than 15 years ago, seemed beyond our grasp. We can construct detailed portraits of how natural selection has acted, and identify variants that increased in frequency as a consequence of positive selection (the process that drives advantageous variants to high frequencies) (reviewed in Fu and Akey 2013). In some cases, it is possible to provide mechanistic links between the favored variant and its phenotypic effect, and to estimate the timescale of selection (for example, in the cases of variants involved in pigmentation (Beleza et al. 2013), lactase persistence (Coelho et al. 2005), and adaptation to altitude (Yi et al. 2010)).

There is also increasing interest in developing methods for the cases in which the advantageous variant was already present in the population at the time of onset of selection (i.e., selection on standing variation) (Messer and Petrov 2013). In addition, methods are being developed to identify instances in which selection favors a combination of genetic variants (polygenic selection), instead of a single advantageous allele (Daub et al. 2013).

Genomic data is helping understand the rate at which we are burdened by deleterious mutations, and the importance of negative selection—which removes deleterious variants from populations—in the human genome (Fu et al. 2013; Henn et al. 2015). Deleterious variants have been hypothesized to play an important role in explaining phenotypic variation, particularly that of common diseases, and population level exome and genome sequencing are being used to tackle this question (with their role remaining controversial, Hunt et al. 2013).

Several studies have also searched for genes under balancing selection, which is the selective regime that maintains several variants in a population at intermediate frequencies, making the persistence time of each allele longer than that of neutral ones. Under this regime, the combination of alleles at a locus is often critical to defining fitness values, and the fitness of an allele may vary over time (reviewed in Key et al. 2014).

Information is also increasingly available for molecular phenotypes, helping understand the functional basis of natural selection. A particularly powerful method is RNAseq, which relies on next generation sequencing of RNA molecules to quantify gene expression. Using such information, Fraser (2013) showed that episodes of recent selection in humans are much more likely to affect gene expression than protein sequence.

Much of the progress in our understanding of how natural selection acts in humans is based on genomewide studies. However, focusing on genes for which we have prior functional knowledge can provide important insights on how natural selection acts. In this review, we integrate knowledge on the function of classical human leukocyte antigen (HLA) genes with population genomic data. We discuss how the genomic perspective both illuminates the study of HLA evolution, and contributes to our understanding of natural selection in the remainder of the genome.

HLA genes code for glycoproteins that bind peptides and present them to T cell receptors. If the bound peptide is non-self (i.e., possibly from a pathogen or a mutated protein), cellular and humoral responses can be mounted (see Box 1). HLA genes also interact with other molecules involved in innate and adaptive immunity. Among these are the killer cell immunoglobulin-like receptors (KIR), for which some HLA class I molecules are ligands (Trowsdale et al. 2001; Parham 2004). When cells are infected or neoplastic, the expression of classical class I loci may decrease, reducing the availability of ligand for KIR molecules. This activates cell lysis by natural killer cells (Yawata et al. 2008).

Research over the last three decades has successfully brought together knowledge on HLA function with advances in theoretical population genetics, allowing evolutionary hypotheses to be tested (in particular through the implementation of neutrality tests, Box 2). There are now several key ideas which are firmly established regarding HLA evolution. First, it is undisputed that HLA genes bear the mark of balancing selection: there are no demographic or genetic factors that can account for the unusually high degree of polymorphism, excess of nonsynonymous variants, or linkage disequilibrium at these genes (Meyer and Thomson 2001; Garrigan and Hedrick 2003; Spurgin and Richardson 2010). Second, there are several lines of support for a role of pathogen-driven selection in shaping HLA variation: HLA genes are associated with susceptibility and resistance to infectious disease (Cagliani and Sironi 2013); experimental studies show that pathogen pressure influences MHC variability (Penn et al. 2002); HLA polymorphism is correlated with pathogen diversity (Prugnolle et al. 2005); variation is highest at sites which define the peptide binding repertoire (Hedrick et al. 1991; Hughes and Nei 1988; Bitarello et al. 2016).

While it is clear that “documenting selection” at HLA genes is no longer a challenge, important questions regarding HLA evolution remain open, and can be addressed using genomic data. First, while it is accepted that balancing selection increases the diversity of HLA genes, there are several types of selection that can produce this effect. Balancing selection is an umbrella term that encompasses heterozygote advantage (or overdominance), selection varying over space or time, and negative frequency-dependent selection (see Box 3). Fleshing out which of these explains the high variability at HLA is a challenge (Spurgin and Richardson 2010), and we discuss the contributions of novel analytical methods and genomewide studies.

Second, the timescale of selection remains an open question. Tests of neutrality used before genomic data became available were only well-powered to detect long-term selection (Garrigan and Hedrick 2003), whereas newer approaches—which rely on dense genetic data spanning thousands of sites—can also detect recent selection (Field et al. 2016; Albrechtsen et al. 2010; Guan 2014). We discuss the findings brought by these approaches, and argue that they indicate that selection on HLA genes can be identified at various timescales.

Third, the increasing understanding of HLA function shows that interactions of HLA genes with other loci—and not just their immediate role in peptide binding—must also be considered in evolutionary studies (Trowsdale and Knight 2013). Further, phenotypic information, including expression levels of the HLA genes, has rarely been incorporated into evolutionary analyses. We discuss the challenges associated to bringing these functional perspectives to the study of HLA evolution.

## HLA variation in the age of genome sequencing

Several generations of methods have been used to identify the alleles carried by an individual: PCR-RFLP, SSOP, immobilized probes, PCR-SSP, and Sanger sequencing (reviewed in Erlich 2012; Carapito et al. 2016). The move to next-generation sequencing (NGS) is actively taking place, and in recent years many protocols have been described for HLA typing and SNP calling (Erlich et al. 2011; Lank et al. 2012; Wang et al. 2012; Danzer et al. 2013; Cao et al. 2013; Major et al. 2013; Langer et al. 2014; Monos and Maiers 2015; Norman et al. 2016; Zhou et al. 2016a).

When deep-sequencing data are available, which is usually the case for HLA-targeted protocols, the tiling of overlapped reads can provide phase information and thus HLA allele sequences (Hosomichi et al. 2013). However, when polymorphisms are on different and non-overlapping reads, statistical approaches to phasing must be used (Castelli et al. 2015, 2017; Lima et al. 2016). Mayor et al. (2015) presented a solution to both the genotype ambiguity and phasing issues by using the PacBio single molecule real time (SMRT) sequencing technology, which generates long reads spanning the entire sequence of individual HLA Class I genes. The method provided accurate and unambiguous HLA genotype calls, representing a promising prospect.

However, an understanding of the role of selection in shaping HLA variation also requires placing it in a genomewide context, so that selective and demographic factors can be disentangled, and genomewide significance testing can be performed. In practice, this requires extracting information on HLA variation from datasets with sequence information for the entire genome. Such data are increasingly generated by exome or whole-genome sequencing, as well as high density SNPs arrays (e.g., The 1000 Genomes Project Consortium 2010; Fu and Akey 2013).

Many genomewide studies, such as Phase I of the 1000 genomes project (The 1000 Genomes Project Consortium 2010), have analyzed HLA polymorphism using standard sequencing pipelines. Given the importance of the 1000 genomes project data to evolutionary research, we previously assessed the reliability of SNP calls which they provide (Brandt et al. 2015). We found that although frequency estimates for HLA SNPs are relatively robust (absolute frequency difference less than 0.1 for 75% of the SNPs), the SNP genotype calls within the HLA loci have alarmingly high error rates (18.6% of calls are incorrect) and are biased toward over-representing the alleles present in the reference genome.

**Fig. 1:**
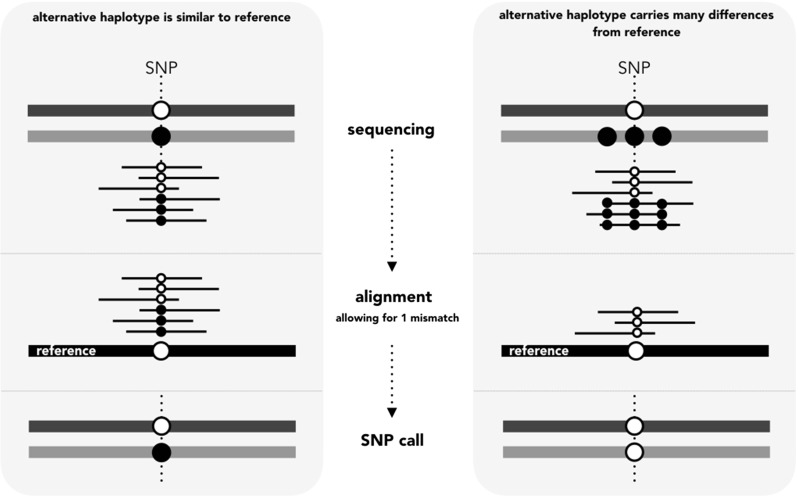
How genotyping errors arise from the mapping of reads to a single reference genome. The *left panel* represents a case where sequence reads come from an individual who is heterozygous at a SNP, but the rest of the gene is similar to the reference for both haplotypes. The reads from both haplotypes can be aligned to the reference, and the SNP genotype is called correctly (i.e., determined by the analysis software). The *panel on the right* shows a case where one of the haplotypes is different from the reference sequence at more positions than the mismatch threshold (in this simple example, only one mismatch is allowed). Reads from this haplotype will not align to the reference sequence and the genotype will be incorrectly called as homozygous at the SNP of interest. Modified from the *Genes to Genomes* blog, http://genestogenomes.org/the-trouble-with-hla-diversity/

This bias occurs because HLA genes are highly polymorphic, and standard methods align short reads (50 to 250 bp) to a single reference genome. Thus, individuals which are heterozygous at a site, but have one allele which is closer to the reference genome, are likely to only map that variant, with the other one failing to align (Fig. 1). The fact that HLA genes are members of a multi-gene family further complicates the sequencing, since reads from one locus can be incorrectly mapped to another.

An increasingly used strategy to address these challenges is to map short reads, generated by NGS, to multiple MHC/HLA references (e.g., IPD-IMGT/HLA database (http://www.ebi.ac.uk/ipd/imgt/hla/)), as opposed to a single reference genome. Recent methods have implemented this idea to efficiently provide more reliable alignments (Castelli et al. 2015, 2017; Lima et al. 2016), HLA allele calls (see Hosomichi et al. 2015 for a review and Bauer et al. (2016) for an evaluation of 12 computational methods), HLA expression estimates (Boegel et al. 2012), or to assemble individual genomes for the MHC region (Dilthey et al. 2015). Encouragingly, Phase III of the 1000 genomes project (The 1000 Genomes Project Consortium 2015) has used this strategy, allowing reads to align to 500 known HLA sequences, in addition to the human reference genome.

A more general solution is to perform genomic alignment using indices which account for the variation across the whole genome, including the MHC. These indices can be built in the form of genome graphs (Novak et al. 2017), an efficient strategy to summarize population-level variation in a graph structure, appropriate for subsequent short-read mapping. Application of such graph indices improves SNP calling in the MHC (Dilthey et al. 2015; Novak et al. 2017), and will likely supplant the use of a single linear reference index in the future. Overall, it appears that a more accurate assessment of HLA variability will come from both the development of new bioinformatic tools, as well as the generation of new data (in particular with long sequencing reads).

Another development is the imputation of HLA alleles based on dense SNP data. Imputation involves using a training set—for which both MHC region SNPs and HLA allele calls are available—to infer the HLA alleles carried by an individual with unknown HLA genotype, but for which SNP data is available (Dilthey et al. 2011; Zheng et al. 2013; Zhou et al. 2016a; Leslie et al. 2008). Zhou et al. (2016a) showed that the concordance rate between imputed HLA alleles and sequencing-based calls can reach 0.93 when using a large reference panel. Imputation is proving to be important in the context of association studies, since it allows an individual’s HLA genotype to be included as a variable (Sanchez-Mazas and Meyer 2014), or even to infer specific amino-acids and amino-acid motifs, and quantify their contribution to overall associations (Jia et al. 2013).

However, imputation-based estimates will be uninformative with respect to novel variants, or those at very low frequencies. When interest is in identifying novel variants (Klitz et al. 2012), deep sequencing associated with mapping methods that account for variation will be required. In addition, imputation accuracy depends on the availability of reference panels with shared ancestry to the target population, representing an important challenge for studies of highly admixed populations with ancestral components which are relatively poorly studied (Levin et al. 2014; Nunes et al. 2016).

In conclusion, we now have access to a wide array of options for uncovering HLA variation. Whereas genomewide sequencing based on alignment to a reference genome generates biased allele frequency estimates, pipelines that account for known HLA diversity can generate accurate information (Dilthey et al. 2015). Importantly, whole genome sequencing places HLA data in a genomewide context, an ideal scenario for separating demographic and selective contributions to variation, as we discuss in the next section.

## Genome scans for balancing selection

The early work on selection at HLA loci was carried out in the “candidate gene” framework, wherein specific HLA loci were tested for selection (see Box 2) (e.g., Hedrick and Thomson 1983, 1986; Hughes and Nei 1988). With genomewide data, on the other hand, it is no longer necessary to *a priori* define which loci will be queried for selection, allowing us to investigate how extreme the evidence for selection at HLA loci is with respect to the remainder of the genome.

Most genomewide scans for selection search for genes that underwent positive selection. The main signatures of this mode of selection are: low variability coupled with extended linkage disequilibrium, caused by the increase in frequency of a favored variant; high population differentiation, due to selection favoring locally adaptive alleles; and an abundance of low frequency variants, due to mutations introducing novel variants into a region recently homogenized by selection (reviewed in Fu and Akey 2013) (see Box 2). Because many of these signatures can also result from non-selective events such as population expansions and bottlenecks, it has become standard for tests of selection to explicitly control for demographic history (e.g., by simulating null distributions under realistic scenarios) (Nielsen et al. 2005). These simulations are parametrized by estimates of the demographic history based on the genomewide data itself. In this way, sets of genes under positive selection have been identified in a robust manner (Akey 2009).

Although there was strong support for positive selection on genes related to immunity (e.g., Nielsen et al. 2005; Tang et al. 2007b; Carlson et al. 2005), few genomic scans found evidence for it in the extended MHC region. Exceptions are the studies of de Bakker et al. (2006) and Sabeti et al. (2006), which identified long range haplotypes in the MHC region. The weak support for selection on HLA genes across several genomewide studies (Akey 2009) is largely a consequence of the fact that they used tests designed to detect positive—and not balancing—selection (Box 2).

In order to detect balancing selection, it is necessary to develop statistics sensitive to deviations expected under this selective regime. Appropriate tests include searching the genome for regions with ancient shared polymorphisms (e.g., Leffler et al. 2013; Teixeira et al. 2015), extreme patterns of polymorphism relative to divergence (e.g., DeGiorgio et al. 2014; Andrés et al. 2009; Bitarello et al. 2017), an excess of intermediate frequency variants (DeGiorgio et al. 2014; Andrés et al. 2009; Bitarello et al. 2017; Hedrick and Thomson 1983), an excess of identity by descent (IBD) (Albrechtsen et al. 2010), or unusually low differentiation between populations (Hofer et al. 2012; Sanchez-Mazas 2007) (Box 2).

Tests using these approaches have been implemented, and the findings for HLA genes are summarized in Table 1. All studies show hits in the MHC region, with *HLA-B* appearing in five out of the six scans (Andrés et al. 2009; DeGiorgio et al. 2014; Leffler et al. 2013; Teixeira et al. 2015; Hofer et al. 2012; Bitarello et al. 2017). In addition, HLA genes show the most extreme evidence of balancing selection in tests based on ancient shared polymorphisms (Klein et al. 1993; Teixeira et al. 2015; Leffler et al. 2013), and are highly enriched for extreme p-values in tests based on polymorphism and divergence (e.g., DeGiorgio et al. 2014; Andrés et al. 2009; Bitarello et al. 2017). This not only confirms that HLA genes have been under long-term balancing selection but also shows that they are extreme in their patterns of diversity, compared to non-HLA loci.

**Table 1:** Findings for HLA genes in genome scans for balancing selection

Reference	Method	Selection timescale^d^	Selection at HLA
Andrés et al. (2009)	SFS and polymorphism/divergence ratio	Ancient	*HLA-B* ^a^
Albrechtsen et al. (2010)	Excess IBD regions^b^	Recent	Entire MHC region
Leffler et al. (2013)	Long-term shared polymorphism	Ancient	*HLA-B* ^c^, *HLA-DQA1*,
			*HLA-DQB1, HLA-DPB1*
DeGiorgio et al. (2014)	Composite likelihood	Long-term	*HLA-A*, *HLA-B*, *HLA-C*,
			*HLA-DRA*, *HLA-DRB1*,
			*HLA-DRB5*, *HLA-DQA1*,
			*HLA-DQB1*, *HLA-DPB1*
Teixeira et al. (2015)	Long-term shared polymorphism	Ancient	*HLA-C*, *HLA-DQA1*, *HLA-DPB1*
Bitarello et al. (2017)	SFS and polymorphism/divergence ratio	Long-term	*HLA-B*, *HLA-C*, *HLA-DPA1*,
			*HLA-DQA1*, *HLA-DPB1*,
			*HLA-DRB1*, *HLA-DRB5*,
			*HLA-DQB2*, *HLA-DQB1*, *HLA-G*

*IBD* identity-by-descent, *SFS* site-frequency spectrum

^a^ Out of five HLA genes analyzed

^b^ A signature compatible with both positive and balancing selection

^c^ The shared polymorphism falling in this gene is a CpG site (has higher mutation rate and could reflect recurrent mutation)

^d^ Long-term: more than 1 million years ago; ancient: greater than species-divergence time (6 million years, for humans and chimps)

The MHC region is also the most extreme in a test based on identity-by-descent (IBD), which identifies genomic regions with extensive identity among individuals, consistent with the hypothesis that they descend from an advantageous ancestral variant (Albrechtsen et al. 2010). This signature supports very recent selection (< 500 generations, or 10,000 years), which can be positive or balancing. Interestingly, Albrechtsen et al. (2010) showed that the increase in IBD is not expected under heterozygote advantage, leading them to argue that selection at HLA loci may be frequency-dependent, or to fluctuate over time, possibly tracking changes in the evolving pool of pathogens that individuals are exposed to.

Important developments in our understanding of HLA evolution have also come from two recent technological breakthroughs: the ability to sequence ancient samples and the genomic analysis of extremely large samples. Using over 200 ancient genomes, Mathieson et al. (2015) found several loci in modern Europeans which experienced greater changes in allele frequencies (with respect to their presumed ancestors, as inferred using the ancient samples), than expected under drift alone. Within the MHC region of Europeans there are at least seven independent signals for selective changes (consistent with both balancing selection or the occurrence of multiple sweeps). New findings also came from the study of Field et al. (2016) which used the theoretical prediction that recently selected variants should be associated with a less diverse genetic neighborhood than the non-selected variants. Leveraged by very large samples of sequence data, they identified genomic regions where selection has driven advantageous alleles to high frequencies in a time frame as recent as 2 000 years, and found that at least three independent SNPs within the extended MHC region were among the most significant targets (Field et al. 2016). This test is designed to detect recent positive selection, implying that balancing selection should not be seen as the only regime relevant to HLA evolution.

Finally, a recent study sequenced genomes of an extant population from the Northwest coast of North America, along with ancient genomes of individuals presumably from the same group, but from before contact with Europeans (Lindo et al. 2016). The study found that at *HLA-DQA1* there was a shift from past positive to recent negative selection, bringing about marked allele frequency changes. The authors conjecture that this may have resulted from environmental or social changes.

In summary, genomic scans for selection have revealed two important patterns. First, when tests designed to identify balancing selection are used, evidence for selection at HLA genes is strong and extreme with respect to the remainder of the genome, confirming what was known based on candidate gene approaches. Second, two studies have identified selection within the MHC region that is consistent with regimes other than heterozygote advantage, and involving very recent time frames (Albrechtsen et al. 2010; Field et al. 2016). According to these studies, and also a recent ancient-DNA study of Lindo et al. (2016), selection drove recent changes in allele frequencies (e.g., via frequency-dependent selection, or selection in a fluctuating selective environment). This supports the view that several selective regimes account for the patterns of variation of HLA genes.

## Disease associations

Identifying HLA variants that contribute to resistance to infectious diseases has important evolutionary implications. Simply put, alleles conferring disease resistance are compelling evidence for past and ongoing selection.

A standard approach for identifying genetic variants that contribute to disease phenotypes is to carry out association studies. These compare the frequencies of genetic variants in groups that differ in a phenotype of interest, such as the occurrence of a specific disease. Thus, for example, if a variant is significantly less common among those with the disease than those without it, it is said to be associated with protection from the disease (provided that case and control groups are carefully controlled for possible confounding variables). Through much of the 1980s and 1990s, HLA variants were tested for association with resistance or susceptibility to infectious diseases. These studies revealed a large number of associations with infectious diseases, some of the most studied being leprosy, malaria, chronic viral hepatitis, and further into the 90s, HIV/AIDS (see Blackwell et al. 2009, for a thorough review). However, these early studies carried important limitations: samples sizes were modest, typically on the order of hundreds, and *a priori* selected candidate genes were investigated, making it difficult to differentiate between associations which were causal or driven by linkage disequilibrium.

The explosion of data that has occurred in the last decade has brought about important changes. Millions of genetic markers are now queried in extremely large samples, allowing genomewide association studies (GWAS) to identify genes or genomic regions associated with diseases, without having to define beforehand the candidate loci to be queried. These association studies are bringing important contributions to our understanding of how genetic variation at HLA genes is related to response to pathogens. Below, we highlight four insights.

First, the recent generation of GWAS have confirmed that variation at HLA genes is directly associated with the outcome of many infectious diseases. Among these are HIV (Fellay et al. 2007), leprosy (Zhang et al. 2009), hepatitis (Kamatani et al. 2009), and tuberculosis (Sveinbjornsson et al. 2016).

Second, diseases which until recently were impractical to study in a GWAS setting can now be investigated. A remarkable example is the analysis led by the personal genomics company 23andMe, which performed an association study for infectious diseases in a sample of 200,000 customers which had volunteered information on various medical conditions (Tian et al. 2016). The study found that variation at HLA genes or within the MHC region is associated with viral (chickenpox, shingles, cold sores, mononucleosis, mumps, warts caused by papillomavirus, strep throat, scarlet fever, pneumonia) and bacterial (tonsil infections, ear infections) diseases.

Third, because GWAS query SNPs throughout the entire MHC region, it is possible to fine-map associations, i.e., identify associations within a narrower region of the genome. This has shown that several associations involve sites with regulatory function. For example, AIDS progression is associated with a 5’ UTR regulatory variant of *HLA-C* (Kulkarni et al. 2011) and hepatitis B recovery is associated with variation at a 3’ UTR site which modulates *-DPB1* expression (Thomas et al. 2012). From an evolutionary perspective, this indicates that selection on HLA genes is not restricted to the structural domains involved in peptide binding, but also involves regulatory variants.

Fourth, dense SNP data allows HLA alleles to be imputed (see Section 3) and thus the amino acid sequence coded by HLA genes to be inferred. In this way, it is possible to study associations at the molecular level, identifying specific changes in a protein that are associated with disease resistance or susceptibility (Nishida et al. 2016; Tian et al. 2016).

Even more activity has taken place in the study of genetic associations with autoimmune diseases. Samples of tens of thousands have routinely been assembled, and copious associations with the MHC region or specific HLA genes have been firmly established, including diabetes, arthritis, celiac disease, lupus, ankylosing spondylitis, multiple sclerosis, psoriasis, and Crohn’s disease (reviewed in Trowsdale and Knight 2013). From an evolutionary perspective, the existence of autoimmune conditions associated with relatively common HLA alleles poses an important question: if the disease reduces an individual’s chances of survival and reproduction, why have the underlying alleles not been driven to low frequencies?

To answer this question, an influential working hypothesis that the same alleles which conferred resistance to infectious diseases and rose in frequency are also associated with autoimmune conditions (Corona et al. 2010; Sams and Hawks 2014; Abadie et al. 2011). This suggests a trade-off occurs, where the benefits brought by disease resistance outweigh the fitness costs of autoimmunity. A formal test involves asking whether alleles that are associated with autoimmune disease risk have increased evidence of having experienced selection. In the context of non-HLA variants, Fumagalli et al. (2011) found a correlation between the abundance of autoimmune disease predisposing variants and pathogen abundance, an indirect support for the trade-off hypothesis. Specifically for HLA, Abadie et al. (2011) examined whether the *HLA-DQA1* variant which predisposes to celiac disease showed evidence of past selection, but found no support. Corona et al. (2010) surveyed GWAS for complex diseases, and found that for type 1 diabetes strongly predisposing SNPs are also those with strong evidence for positive selection.

Although this approach has not yet delivered a clear picture, the strong evidence of pathogen-driven selection at HLA genes, coupled with the extreme abundance of HLA involvement in autoimmunity, call for further development of evolutionary approaches investigating the possibility that there is a causal connection between evolutionary response to infectious diseases and autoimmunity.

## Multilocus effects: epistasis and hitchhiking

There is increasing awareness that many adaptive traits are polygenic, and that searching for allele frequency changes at multiple loci is an important improvement over “single locus” approaches (Daub et al. 2013; Berg and Coop 2014). There are several reasons why we expect adaptation involving HLA genes to be polygenic, which we discuss below.

There is support for epistatic interactions between variants at distinct HLA loci, driving advantageous haplotypes to higher frequencies than expected by chance, and thus explaining the high linkage disequilibrium in the MHC. One reason why a haplotype may be favored is that it carries a combination of alleles that presents a broader range of pathogenic peptides than expected for a random pair of alleles. This hypothesis was recently supported by a theoretical model, as well as data analyses showing that alleles in linkage disequilibrium on average have a lower overlap in the peptide binding repertoire than expected by chance (Penman et al. 2013). Using a simulation-based approach, van Oosterhout (2009) also illustrated that epistasis among HLA loci can play an important role in shaping extant patterns of diversity. Finally, GWAS for HLA loci found multi-locus effects, as is the case of the association of the *DR2* haplotype (DRB1*1501 and DRB5*0101) with multiple sclerosis (Gregersen et al. 2006).

Second, multi-locus interactions have also been documented between HLA genes and those outside the extended MHC (see Box 1). For example, Kirino et al. (2013) found a strong epistatic interaction between HLA-B*51 and the *ERAP1* locus, with one specific genotype greatly increasing the susceptibility to Behçet’s disease. *ERAP1* codes for the protein responsible for trimming the pathogens to be loaded and presented by HLA class I molecules, making interactions between it and HLA genes functionally plausible.

Another case of epistasis involves the interaction between HLA and KIR. KIR molecules can recognize HLA class I molecules carrying HLA-A3, -A11, -Bw4, -B27, -C1, or -C2 epitopes, as well as *HLA-F* and possibly *HLA-G* (reviewed in Parham et al. 2012). In a study of 30 human populations, Single et al. (2007) found a strong negative correlation between the frequency of *HLA-B* alleles of the *Bw4* group, which carry an isoleucine at position 80, and the presence of *KIR3DS1* gene. Because *Bw4* alleles are ligands for *KIR3DS1*, which is an “activator” (a gene whose protein product initiates a cytotoxic response), the combination of high frequencies of ligand and receptors would result in an abundance of excessively activating genotypes, which are prone to autoimmunity. At the other extreme, combinations of low frequencies of ligand and *KIR3DS1* would result in an excessively weak KIR response, increasing the susceptibility to infection. Selection against genotypes at these extremes could account for the observed correlations seen in Single et al. (2007). Using a similar approach, Hollenbach et al. (2013) found strong (*r* > 0.79) and significant correlations between the frequencies of *KIR2DL3* and HLA-C1 in 45 populations.

Support for these interactions also comes from the study of specific populations. In the African KhoeSan, the C2 allotype occurs at an unusually high frequency (63%), whereas in the Yucpa of South America it is the C1 allotype that is common (83%) (Hilton et al. 2015; Gendzekhadze et al. 2009). Strikingly, in both populations, the receptors for these common allotypes show evidence of having been recently selected and driven to high frequencies, with the mutant forms having reduced or complete lack of function. In both cases, these population-specific variants may have been favored due to their ability to restore a balance between C1, C2, and the KIR inhibitory allotypes, providing the benefits of reducing the chances of originating preeclampsia predisposing genotypes (see below). Functional studies provide further support for epistasis, showing that homozygotes for HLA-C1 respond more intensely to a viral infection than those carrying HLA-C2 alleles (Ahlenstiel et al. 2008, see also Augusto et al. 2015, for an example involving the autoimmune disease pemphigus).

The epistatic interactions between KIR and HLA also influence reproduction. For example, mothers homozygous for the KIR haplotype from group A (defined by the presence of four framework genes—*KIR2DL4*, *KIR3DL2*, *KIR3DL3*, and *KIR3DP1*—and *KIR2DL1*, *KIR2DL3*, *KIR2DS4*, and *KIR3DL1*) have an increased rate of miscarriage, pre-eclampsia, and weight restriction at birth when they also carry an HLA-C1 allele and the fetus has an HLA-C2 allele. This results from a less effective remodeling of blood vessels, necessary for placentation (Penman et al. 2016; Hiby et al. 2014; Hiby et al. 2004). On the other hand, individuals with group A KIR haplotypes and HLA-C1 alleles respond to viral infections more efficiently than individuals with group B haplotypes (which carry genes encoding KIRs with decreased or no binding to HLA class I molecules, such as *KIR2DS2*, *KIR2DS3*, and *KIR2DS5*) in combination with HLA-C2 alleles (e.g., hepatitis C and HIV clearance). This tradeoff may result in alternating episodes of reproductive and pathogen-driven selection, explaining the maintenance of polymorphism for KIR haplotypes and for the HLA-C1 and -C2 group alleles in many human populations. This scenario was supported by computer simulations (Penman et al. 2016) and is consistent with patterns of HLA and KIR polymorphism in many human populations (see details in Trowsdale and Moffett 2008; Parham and Moffett 2013; Augusto and Petzl-Erler 2016).

Strong selection at a locus can also influence variation at linked sites through genetic hitchhiking. Under pathogen- driven selection an advantageous variant is driven to higher frequencies at a greater speed than would be expected under drift, and can thus drag linked variants (Charlesworth 2006). This selective regime can increase the frequency of slightly deleterious mutations near the selected gene. Accordingly, Chun and Fay (2011) showed that for regions in the neighborhood of sites with strong evidence for positive selection, there is an enrichment for deleterious polymorphism.

**Fig. 2:**
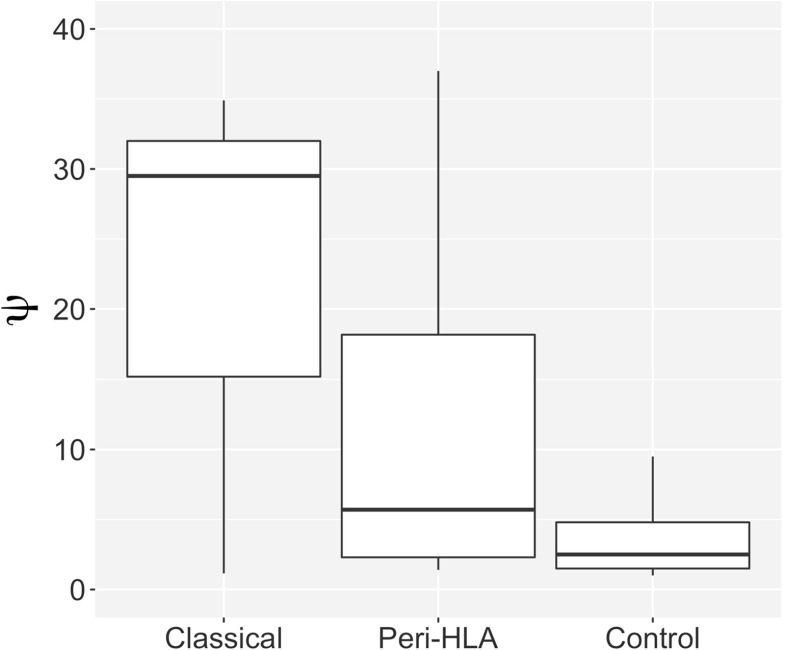
The value of *ψ*, a statistic that measures the proportion of deleterious variants, in three sets of SNPs. The statistic is defined by $\psi = \frac {L_{S}.P_{N}}{L_{N}.(P_{S}+1)}$, where *P* represents the number of polymorphic sites, *L* represents the number of potentially mutable sites, and *S* and *N* subscripts refer to synonymous and nonsynonymous sites. Higher values of *ψ* indicate a greater proportion of deleterious (or functional, in the case of the SNPs from the classical HLA genes) variants. Values are shown for exons of classical HLA genes, genes in the immediate neighborhood of the HLA genes (“peri-HLA”), and genes outside the MHC region. Values were computed for sites with a minor allele frequency (MAF) greater than 0.05, to avoid the effect of rare deleterious variants, which are overrepresented in the control set. The peri-HLA genes have higher load (*ψ*) than the controls

In the context of the MHC region, a natural hypothesis is that genes close to the classical HLA loci will show an enrichment of deleterious variants, with respect to the expectations based on genomewide controls. Mendes (2013) investigated this hypothesis, and in an analysis of the 1000 Genomes data (The 1000 Genomes Project Consortium 2010) found that genes that hitchhike with HLA loci have an increased proportion of putatively deleterious variants (Fig. 2). This hypothesis was also tested by Lenz et al. (2016), who used a larger exome-based dataset to show an excess of intermediate frequency deleterious polymorphism within the MHC. Further, these authors used simulations to show that strong balancing selection—comparable in strength to that seen at HLA genes—makes deleterious variants more common than would be expected without the hitchhiking effect.

These findings are particularly important given the large number of disease associations in the MHC region (including the flanking non-HLA loci), suggesting that balancing selection in HLA genes may drive the accumulation of deleterious variants in their neighborhood, contributing to the associations with disease phenotypes.

To conclude, we emphasize that ongoing research recommends that variation at HLA genes be studied with reference to both the genes they interact with, as well as considering how physical linkage leads to changes in polymorphism at neighboring sites. Placing HLA variation in a genomewide context will be essential in order to achieve these goals.

## Population differentiation

If distinct populations are under a regime of selection favoring HLA heterozygotes, population differentiation, measured by *F*_*ST*_, is expected to be lower at HLA than at neutral loci (Schierup et al. 2000). This is because balancing selection maintains alleles segregating in populations for longer than expected under neutrality, reducing *F*_*ST*_ (Box 2).

An alternative scenario is that selection favors different alleles in distinct populations, driving locally adaptive HLA alleles to higher frequencies, increasing population differentiation.This expectation is consistent with pathogen-driven selection at HLA, for which there is theoretical (Borghans et al. 2004; Hedrick 2002) and empirical support (e.g., Prugnolle et al. 2005; Hedrick 2006). Given the premise that pathogen populations differ between regions, pathogen-driven selection could drive locally adaptive HLA alleles to higher frequencies, and thus cause an increase in population differentiation.

Surprisingly, support for both of these markedly different expectations has been found (Table 2), with some studies showing HLA to be unusually highly differentiated, and others reporting unusually low differentiation at HLA. What is the cause for the inconsistency among studies? Analyses using *F*_*ST*_ are sensitive to various aspects of the methodology, all of which can influence the results, as we discuss below.

**Table 2:** Population differentiation at HLA genes relative to neutral markers

Reference	Neutral marker	HLA marker	Method	*F*_*ST*_ in HLA
Akey et al. (2002)	SNP	SNP (genomewide scan)	Empirical outlier	Not an outlier
Meyer et al. (2006)	Microsatellites	HLA allele^a^	Empirical outlier	Not an outlier
Sanchez-Mazas (2007)	Microsatellites and RFLPs	HLA allele^a^	Empirical outlier	Lower in HLA
Bhatia et al. (2011)	SNP	SNP (genomewide scan)	Tree-based test	Higher in HLA
Nunes (2011)	Microsatellites	Microsatellites	Simulation	Higher in HLA
Hofer et al. (2012)	SNP	SNP (genomewide scan)	Simulation	Lower around *HLA-C*
Colonna et al. (2014)	SNP	SNP (genomewide scan)	Empirical outlier + clustering	Not an outlier
Brandt (2015)	SNP	SNP and HLA alleles	Empirical outlier	Lower for HLA SNPs; HLA alleles are not outliers

^a^ See Box 1 for the definition of HLA allele

First, studies which compare different markers, such as HLA alleles and microsatellites, are sensitive to the effects of the mutational mechanism and mean heterozygosity on *F*_*ST*_, making direct contrasts between HLA and non-HLA markers unreliable (a challenge for the studies of Meyer et al. 2006; Sanchez-Mazas 2007). Second, the statistical tests used to define extreme *F*_*ST*_ differ among studies, including outlier approaches, tree-based tests, simulation under an various demographic models, among others (Table 2). Third, the power to detect balancing selection may vary depending on the timescale of separation of populations, and features of their demographic histories (reduced HLA differentiation being harder to detect in admixed populations, for which genomewide *F*_*ST*_ is lower). Fourth, SNPs with low heterozygosities are constrained to low *F*_*ST*_, implying that HLA and non-HLA SNPs must be compared in a way that accounts for this effect (Bhatia et al. 2013).

**Fig. 3:**
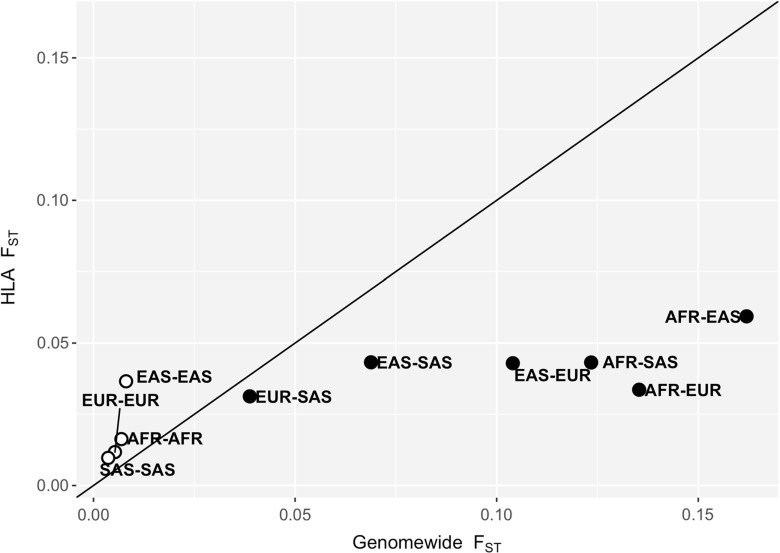
*F*_*ST*_ among pairs of populations. Each point depicts the mean *F*_*ST*_ for non-HLA (*x*-axis) and HLA (*y*-axis) SNPs between pairs of populations in each continent (AFR: Africa; EAS: East Asia; EUR: Europe; SAS: Southeast Asia). Pairs of populations from the same continent are represented by white-filled points, and pairs of populations from different continents, by *solid black points*. SNP data was acquired from the 1000 Genomes data phase III (The 1000 Genomes Project Consortium 2015), and HLA SNPs were filtered according to Brandt et al. (2015) to avoid errors due to mapping bias. *F*_*ST*_ values were weighted by allele frequency, so that the excess of rare variants in the non-HLA SNPs does not cause a reduction of mean *F*_*ST*_ in that class. Notice that HLA differentiation is higher than genomewide for population pairs from the same continent, and lower than genomewide when populations from different continents are compared

In order to overcome these issues, we analyzed HLA differentiation among major continental groups, accounting for these effects (Brandt 2015) (Fig. 3). Marker-type effects are accounted for by only analyzing SNP data. The non-HLA SNPs provide expectations due to demographic processes, allowing a statistical assessment of how extreme the differentiation is for SNPs within HLA loci. *F*_*ST*_ values for SNPs in the HLA and non-HLA groups are averaged using an approach that controls for the differing heterozygosity distributions in those groups (Reynolds et al. 1983; Bhatia et al. 2013). With these methodological controls in place, the results in Fig. 3 show that SNPs within HLA genes have lower *F*_*ST*_ than genomewide SNPs when we compare highly diverged populations (i.e., those from different continents). Population pairs from the same continent have higher differentiation for SNPs in the HLA genes compared to other genomic regions.

How do these findings compare to those of previous studies? Low differentiation among HLA SNPs is consistent with the findings of Hofer et al. (2012), which detected a similar pattern in a dataset including highly divergent human populations. The increased differentiation seen by Bhatia et al. (2011) among African populations is also consistent with this result, since that study analyzed closely related populations.

Also, one of the SNPs driving the high differentiation reported in Bhatia et al. (2011) was linked to *HLA-DPA1*, a locus we excluded because it did not show strong evidence of balancing selection in previous studies (Solberg et al. 2008; Begovich et al. 2001), and showed instances of directional selection (Hollenbach et al. 2001). Indeed, for *HLA-DPA1* population differentiation was higher than genomewide in our data as well, consistent with local positive selection. Interestingly, *HLA-DPA1* has one of the strongest signatures of long term balancing selection in Bitarello et al. (2017). A plausible scenario is that *HLA-DPA1* is under a selective regime that varies through time, leaving a signature of past balancing selection and more recent local positive selection.

Given the overall result that natural selection on HLA genes, over long periods of time, results in decreased population differentiation (Fig. 3, *y*-axis), it is natural to consider how to reconcile this with the expectation that pathogens would drive local adaptation, making populations more different from one another at HLA genes. There are two possible ways in which low differentiation at HLA SNPs can be reconciled with a model of local adaptation of HLA alleles.

First, the signal of local adaptation (high differentiation) may only be detectable when comparing closely related populations, such as the ones in the same continent. Indeed, previous studies have detected high differentiation in HLA alleles between populations within the same continent (Cao et al. 2004; Qian et al. 2013), and we have detected higher *F*_*ST*_ at HLA SNPs than genomewide SNPs for pairs of populations in the same continent (Fig. 3).

Second, low differentiation at SNPs and high differentiation at HLA alleles may be expected if we consider that HLA alleles are defined by multiple SNPs, and that most SNPs are shared between two or more alleles. The important role that intragenic recombination and gene conversion play in generating HLA allele diversity also contributes to the sharing of SNPs among different HLA alleles (Parham and Ohta 1996). Thus, a plausible scenario is that individual SNPs have low *F*_*ST*_, but the haplotypes which they define may show high divergence. Biologically this amounts to considering that balancing selection favors the maintenance of polymorphism at specific sites, key to defining peptide binding specificities (Bitarello et al. 2016). However, the specific combinations of variants (i.e., the HLA alleles) that become more frequent differ among populations as a function of the pathogens driving the selection.

## Selection and admixture

Individuals in admixed populations have genomes which are a mosaic of different ancestries (Winkler et al. 2010). The size and ancestry of segments is determined by factors which are demographic (e.g., proportion of ancestors from each ancestry, timing of admixture) and genetic (e.g., recombination rates). If genetic variants from one of the parental populations are advantageous to individuals in the admixed population, they will rise in frequency and thus cause an over-representation of a specific parental ancestry in the genomic region under selection. Thus, regions of the genome exhibiting ancestry proportions that deviate from the genomewide average provide evidence for recent selection.

**Fig. 4:**
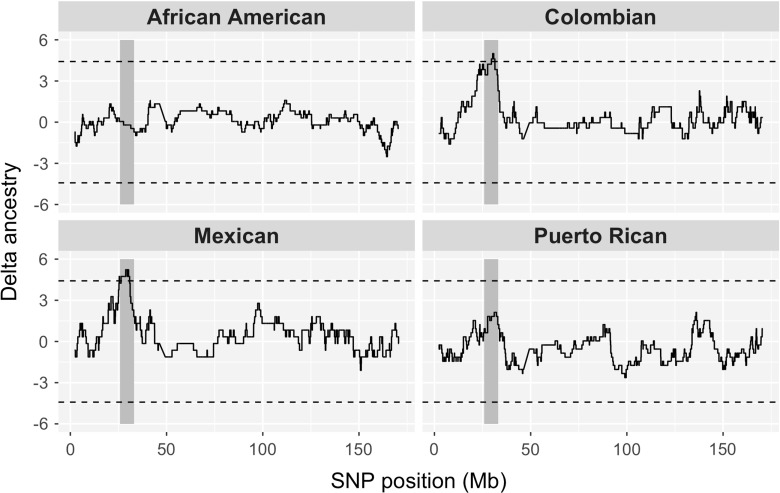
Deviation from average genomewide ancestry in four admixed populations along chromosome 6. The degree to which local ancestry deviates from genomewide averages is shown for African ancestry (*black lines*). The region encompassing the MHC region is indicated by *gray shading*. Ancestral and admixed populations are from the 1000 genomes project (African and European; ftp://ftp.1000genomes.ebi.ac.uk/vol1/ftp/release/20110521/), except for the ancestral Native American sample, which is from the HGDP-CEPH (http://www.cephb.fr/hgdp/index.php). Local ancestries were estimated using RFMIX (Maples et al. 2013). The ancestry deviation measure is the difference between ancestry at a given genomic position with respect to the genomewide average, normalized by the standard deviation of the ancestry estimate (thus providing a measure of the number of standard deviations each ancestry departs from its genomewide average)

To illustrate the power of this approach in understanding selection at HLA genes, we calculated local ancestries (i.e., the ancestry of a specific position of the genome) for individuals from four admixed populations (The 1000 Genomes Project Consortium 2010). For each position in the genome, we quantified how much the ancestry proportions differed from the genomewide average, within each population. For chromosome 6, we find that two of the populations (Colombian and Mexican) have an excess of African ancestry in the MHC region (the threshold of significance set at 4.4 standard deviations, following Seldin et al. 2011) (Fig. 4).

To explore this pattern further, we reviewed the findings of eight studies that investigated the distribution of local ancestries and recorded how often the MHC showed unusual ancestry proportions with respect to genomewide averages. In total, six out of eight studies report an excess of African ancestry in the MHC region for at least one admixed population (Table 3). Interestingly, this effect is seen in populations with different admixture histories, distinct African parental populations and proportion of contributions, and using different methods to estimate local ancestry. Overall, the support for deviation in local ancestry for the MHC region is strong and recurrent, prompting us to consider both its possible biological basis as well as the likelihood of methodological artifacts.

**Table 3:** Ancestry proportions in the MHC region vs genomewide

Reference	Admixed population	Method	Observation
Tang et al. (2007a)	Puerto Rican	Frape	Excess African
Johnson et al. (2011)	Mexicans	SABER +	Excess European
Brisbin et al. (2012)	Four Latino populations^a^	PCAdmix	Excess African in Colombian, Puerto Rican and Ecuadorian
Bhatia et al. (2014)	African Americans	RFmix	non-significant increase in African
Guan (2014)	Mexican	ELAI	Excess African
Rishishwar et al. (2015)	Colombian	SUPORTMix	Excess African
Zhou et al. (2016b)	Mexican	ELAI	Excess African
Deng et al. (2016)	Seven Latino populations^b^	Structure and Z-test	Excess African

^a^ Dominican Republic, Colombian, Puerto Rican, Ecuadorian

^b^ Mexican, Guatemalan, Costa Rican, Colombian, Chilean, Argentinean, Brazilian

A basic concern is whether local ancestry methods are biased by features of the MHC region (other than a true shift in ancestry proportions). For example, Price et al. (2008) pointed out that most deviations in ancestry reported by Tang et al. (2007a) (both within and outside the MHC region) were associated with regions of high linkage disequilibrium (LD). However, new methods for detecting local ancestry control for LD, but still detect an excess of African ancestry in the MHC (Guan 2014; Brisbin et al. 2012) (Table 3). An additional concern is that some ancestry inference methods require phased data, something that is challenging for the MHC, given the high polymorphism. However, ancestry results are consistent across methods that do (e.g., Brisbin et al. 2012) and do not (Guan 2014) require phased data, suggesting this is not the factor driving the findings.

Further problems for local ancestry estimation were raised by Pasaniuc et al. (2013), who found that loci with increased deviation in local ancestry show high polymorphism and increased rates of mendelian inconsistency. These authors also showed that inappropriate parental reference panels (e.g., distantly related from the true parental populations) can introduce errors in the analysis. This fact is of extreme relevance since samples from the true parental populations are not always available.

Further studies will be needed so as to evaluate whether technical artifacts underlie the shifts in ancestry proportions in the MHC region. In this sense, a promising result was reported by Deng et al. (2016), who used simulations under a human demographic model to show that the ancestry deviation in the MHC of Latin American populations is not expected in the absence of selection. In addition, Tang et al. (2007a) showed that an unusual African ancestry proportion in the MHC region of Puerto Rican individuals is found using local ancestry analysis based on SNPs, as well as more traditional admixture estimates using classical HLA markers and microsatellites, providing additional evidence that the shifts in ancestry are not a feature observed with one type of marker or inference method.

Ancestry deviations place the MHC as a striking example of a genomic region under strong recent selection. Nevertheless, even if this general picture is confirmed in new studies, several questions remain to be addressed. First, how many and which HLA alleles are favored by selection, causing the deviation in local ancestry? Second, is the recurrent finding of excess African ancestry explained by higher genomewide diversity in Africans (which indirectly could lead to the harboring of more advantageous variants)? Clearly, a biological understanding of these patterns is still lacking.

Selection favoring alleles of a specific ancestry can also be seen through the analysis of archaic genomes. These studies found evidence for adaptive introgression from archaic groups (Denisova and Neanderthal) into modern humans (reviewed in Racimo et al. 2015), including in the MHC region. Abi-Rached et al. (2011) suggested that a highly divergent allele, HLA-vB*73, entered the modern human gene pool through introgression from archaic hominins. In modern populations, HLA-B*73 is practically absent everywhere except West Asia, and almost all haplotypes carrying HLA-B*73 also carry HLA-C*15:05, which only reaches appreciable frequencies in Asia (Abi-Rached et al. 2011). Simulations showed that introgression from archaic hominins provides a better fit to the data than a model in which the allele arose in Africa before the Out-of-Africa event (Abi-Rached et al. 2011).

Yasukochi and Ohashi (2016) argue that this evidence is circumstancial, noting that B*73 was not found in any archaic genome and that strong long-term balancing selection could maintain the alleles independently in both species. Also, if Denisova introgression into modern humans occurred in Southeast Asia, that is where HLA-B*73 should have higher frequency.

On the other hand, Abi-Rached et al. (2011) found even more compelling evidence for adaptive introgression coming from the HLA-A*11 allele, which occurs at high frequencies in Papua New Guinea and China (but is absent from Sub-Saharan African) and is found in long haplotypes with HLA-C*15 and HLA-C*12, both of which exhibit higher diversity in Asia than in Africa. A likely explanation is that all HLA-A*11 found in modern humans came from Denisovan introgression, followed by a rise in frequency in Asia. In brief, it may well be that when humans left Africa, they encountered new selective pressures to which archaic hominins were better adapted on a local scale, and strong selection favored those adaptive variants acquired through introgression. However, current evidence for adaptive introgression of HLA alleles should be interpreted with caution because of the technical difficulties in assessing variability of HLA genes, and small sample sizes of archaic species. Also, apparent introgression might result from incomplete lineage sorting, which is particularly likely in the MHC region, where long-term balancing selection results in trans-specific polymorphisms (Klein et al. 1993; Teixeira et al. 2015; Leffler et al. 2013).

## From genome to transcriptome

While most studies on selection at HLA genes focus on peptide binding properties, expression levels are also important in determining phenotypes related to disease progression, both for infection and cancer (Blais et al. 2012; Thomas et al. 2012; Apps et al. 2013; Boegel et al. 2014). For example, high expression of *HLA-C* enhances an individual’s ability to respond to HIV infection, whereas low expression confers protection against Crohn’s disease (Blais et al. 2012; Apps et al. 2013). Additionally, expression varies broadly among tumor types, ranging from loss/downregulation to high expression (Boegel et al. 2014). Such opposing effects of expression levels may account for the selective maintenance of differential expression across HLA alleles or haplotypes.

Despite the potential importance of HLA expression to evolutionary and medical studies, few datasets with this information have been generated. To a large degree, this results from the the difficulty in quantifying expression for genes which show an unusually high polymorphism and are members of a multi-gene family. For highly polymorphic genes, array-based expression requires probes that avoid polymorphic regions, which if not accounted for can cause differential binding due to genetic variation, biasing expression estimates. The same difficulty applies to quantitative PCR, which needs primers that can bind the entire range of alleles of a specific locus, posing an important challenge when developing the experimental design.

To overcome these difficulties, customized arrays (Vandiedonck et al. 2011) and qPCR primer sets (Ramsuran et al. 2015) have been developed. These account for polymorphism and can provide locus-level expression estimates. However, these studies are limited in the number of samples and population diversity surveyed, and the requirement of custom arrays or primer sets makes repetition of surveys on additional populations and extension to other HLA loci challenging. Further, the expression of each allele cannot be directly estimated, and is instead imputed from the locus-level expression of homozygotes (Ramsuran et al. 2015). This places the quantification of HLA expression as an enterprise still in its infancy, although the studies carried out to date show that HLA expression varies between alleles, loci, and tissues (Boegel et al. 2012; Boegel et al. 2014; Ramsuran et al. 2015; Melé et al. 2015).

The RNAseq technology, which quantifies expression using NGS, is increasingly being used in genomewide studies and has the potential to provide large-scale information on HLA expression, but also has challenges. The technology relies on the mapping of short reads (generated by sequencing the transcriptome) to an index, so as to quantify the abundance of mRNA originating from each gene or exon. In the event that the surveyed individual is highly divergent from the sequences in the index (as is often the case due to the high polymorphism of HLA genes), it is likely that many reads will be discarded due to large numbers of mismatches, failing to document expression, and biasing the estimates toward the overexpression of variants which are more similar to the one in the index. This results in inaccurate and/or biased gene expression estimates and can cause spurious eQTLs to be identified (Panousis et al. 2014). This problem is similar to that of read mapping for HLA genes in NGS, discussed in Section 3 (Brandt et al. 2015).

As a consequence, large studies which surveyed the whole-transcriptome in many individuals (e.g., Lappalainen et al. 2013; Battle et al. 2014) using high-throughput technologies do not provide reliable estimates for the expression of HLA genes. An alternative is the development of bioinformatic tools that use whole-transcriptome RNAseq data to accurately estimate HLA expression. This has the benefit of placing the HLA expression data within the context of genomewide expression levels, and allows the use of RNAseq datasets that are already available (Lappalainen et al. 2013; Battle et al. 2014; Melé et al. 2015).

A promising approach is to use of an index with thousands of HLA sequences reported in databases such as IPD-IMGT/HLA, instead of relying on a single reference genome. For example, *seq2HLA* is a pipeline proposed by Boegel et al. (2012) which uses a form of *in silico* genotyping to both infer the genotypes at HLA genes as well as estimate the expression of each HLA allele at a locus. Such allele-specific estimates are not obtained when RNAseq data is processed by standard pipelines, which provide expression estimates at the level of genomic features such as annotated genes, exons or isoforms.

**Fig. 5:**
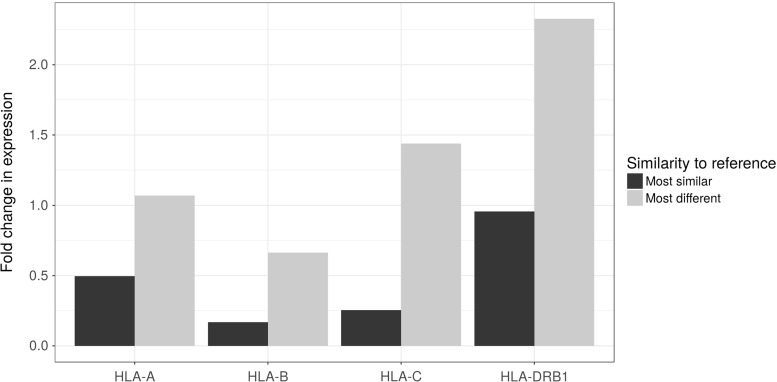
Fold change in expression estimates obtained by kallisto (Bray et al. 2016) using a supplemented index relative to a standard reference index (*y* = 0). Results are presented for genotypes with different degrees of similarity to the reference genome (*bar colors*). We used 48 CEU individuals for which RNAseq data are available from the Geuvadis consortium (Lappalainen et al. 2013) and HLA genotypes were determined by Sanger sequencing (Gourraud et al. 2014). Genotypes at each locus were divided according to quartiles of differences from the reference allele at that locus. “Most similar” and “Most different” correspond to the first and fourth quartiles respectively (12 individuals each).

The work by Boegel et al. (2012) showed that the use of an appropriate index (i.e., the set of reference sequences to which the short reads generated by the NGS will be aligned) is the key element for the improvement in the estimates. The benefits of this approach are shown in Fig. 5: expression estimates increase when using indices supplemented with many HLA sequences, relative to expression estimated using the single reference genome. This effect is more pronounced for individuals carrying alleles which are most different from the reference. This is expected, since these are the cases where the use of the reference genome leads to the greatest underestimation of expression.

This result suggests that bioinformatic methods tailored to deal with HLA diversity can bring important changes to expression estimates and thus to eQTLs mapped, providing new hypotheses for functional elements which drive HLA expression variation. Promising candidates will include UTR sites, promoter/enhancer polymorphism, transcription factor binding sites, etc, all of which have been documented as enriched categories of eQTLs in standard genomewide studies (e.g., Lappalainen et al. 2013).

It will also be possible to further explore initial findings regarding expression differences among genes and alleles (revealed by qPCR studies). In particular, the pattern of relatively even expression among *HLA-B* alleles (Ramsuran et al. 2017), and variable expression levels among lineages at *HLA-A* (Ramsuran et al. 2015) and *HLA-C* (Apps et al. 2013) will be amenable to investigation on a wider scale.

## Conclusions

Our current knowledge of HLA evolution differs with respect to that of a decade ago in many ways. To a large degree, this results from our ability to place HLA variation within the context of the entire genome. Genomewide studies have contributed to our understanding of selection by increasing the power of tests (thanks to the large number of samples and genetic markers) and by allowing variation from the entire genome to be used as a control for complicating factors, including population history. We now have evidence that selection on classical HLA genes extends beyond the heterozygote advantage model and has operated from ancient to very recent timescales (Albrechtsen et al. 2010; Field et al. 2016; Tang et al. 2007a; Mathieson et al. 2015).

By comparing genetic differentiation at HLA genes to that of the remainder of the genome, we have found instances of decreased differentiation (e.g., Hofer et al. 2012), as well as of increased differentiation (Bhatia et al. 2011). Such studies will help investigate which HLA variants represent adaptations to local selective pressures, and which are shared extensively at global scale, as an outcome of long-term balancing selection. We are now also able to investigate patterns of admixture in HLA genes (Tang et al. 2007a; Guan 2014), providing insights into the time frame and mode of selection that occurs when populations of different ancestries meet and interbreed.

We can increasingly test co-evolutionary hypotheses, such as the relation between KIR and HLA polymorphism (e.g., Single et al. 2007), and test hypotheses of epistatic interactions. Genomic data also allows us to test the effect of strong selection on HLA upon linked variants, a process which may be driving the accumulation of deleterious mutations near HLA genes (e.g., Lenz et al. 2016).

A whole new layer of information, namely expression levels, can be generated on a large scale, and integrated with information on genetic variation. This will contribute to association studies, by incorporating a key cellular phenotype—expression level—as a covariate. Such approaches will also help bring functional information to the investigation of HLA evolution (for example, in the form of allelic lineages (Bitarello et al. 2016) or supertype grouping (Francisco et al. 2015)).

Our perspective is that, increasingly, we will see the immunogenetics community working closely with researchers in genomics. Placing HLA within the genomic context is key to understanding HLA genes; complementarily, immunogenetics expertise will be key to interpreting genomewide studies, within which HLA genes are frequent and striking findings (be it in GWAS, selection, admixture or expression studies). In addition, lessons and challenges associated with studying a highly polymorphic region under intense balancing selection, as is the case for the MHC, can be carried over to the study of other genes or genomic regions under balancing selection (Leffler et al. 2013; Teixeira et al. 2015; DeGiorgio et al. 2014; Bitarello et al. 2017).
